# Electrical Detection of Charge-to-spin and Spin-to-Charge Conversion in a Topological Insulator Bi_2_Te_3_ Using BN/Al_2_O_3_ Hybrid Tunnel Barrier

**DOI:** 10.1038/s41598-018-28547-y

**Published:** 2018-07-06

**Authors:** C. H. Li, O. M. J. van ‘t Erve, C. Yan, L. Li, B. T. Jonker

**Affiliations:** 1 Materials Science and Technology Division, Naval Research Laboratory, Washington, DC 20375 USA; 2 Department of Physics and Astronomy, West Virginia University, Morgantown, WV 26506 USA

## Abstract

One of the most striking properties of three-dimensional topological insulators (TIs) is spin-momentum locking, where the spin is locked at right angles to momentum and hence an unpolarized charge current creates a net spin polarization. Alternatively, if a net spin is injected into the TI surface state system, it is distinctively associated with a unique carrier momentum and hence should generate a charge accumulation, as in the so-called inverse Edelstein effect. Here using a Fe/Al_2_O_3_/BN tunnel barrier, we demonstrate both effects in a single device in Bi_2_Te_3_: the electrical detection of the spin accumulation generated by an unpolarized current flowing through the surface states, and that of the charge accumulation generated by spins injected into the surface state system. This work is the first to utilize BN as part of a hybrid tunnel barrier on TI, where we observed a high spin polarization of 93% for the TI surfaces states. The reverse spin-to-charge measurement is an independent confirmation that spin and momentum are locked in the surface states of TI, and offers additional avenues for spin manipulation. It further demonstrates the robustness and versatility of electrical access to the spin system within TI surface states, an important step towards its utilization in TI-based spintronics devices.

## Introduction

A bulk topological insulator (TI) is a new quantum phase of matter characterized by an insulating bulk, and gapless surface states that are occupied by massless Dirac fermions which exhibit many intriguing properties^1–5^. Spin-momentum locking is one of the most remarkable properties of TI, where the surface state spin lies in plane and is locked at 90 degrees to carrier momentum, so that an unpolarized charge current creates a net spin polarization. This property has been extensively probed by surface sensitive techniques such as spin and angle resolved photoemission spectroscopy^6–8^, as well as polarized optical spectroscopy^9^. We recently demonstrated the first detection of current induced spin polarization in the surface states of a topological insulator in a transport geometry, using a ferromagnet/tunnel barrier detector contact, where the projection of the TI spin onto the detector magnetization was measured as a voltage^10^. Current-generated spin polarization has also been observed in other TI systems^11–17^. We have further shown that the current-induced spin polarization in the TI Dirac surface states is opposite to that of the trivial two-dimensional electron gas states^18^, and in the case when these two states coexist on the TI surface, as in degenerate Bi_2_Se_3_ with band bending at the surface, the TI Dirac surface state spin polarization dominates, consistent with theoretical predictions^19^.

Conversely, if spin-polarized carriers are injected into the Dirac surface states, spin-momentum locking dictates that carriers with a distinct spin orientation should exhibit a particular momentum, or direction of motion. Hence a charge accumulation should be generated in the direction orthogonal to the injected spin polarization, referred to as the inverse Edelstein effect^20^. Such spin-to-charge conversion has been demonstrated in a range of TIs including chalcogenides^13,21–23^ and alpha Sn^24^, as well as the topological Kondo insulator SmB_6_^25^. In most cases, spin injection was accomplished by spin pumping via ferromagnetic resonance from a ferromagnetic contact.

In this work, we demonstrate both spin-to-charge and charge-to-spin conversion *electrically* in the same device for the TI Bi_2_Te_3_. Using an Fe/Al_2_O_3_/BN hybrid tunnel contact, we electrically detect the spin accumulation generated by an unpolarized current flowing through the Dirac surface states, and estimate a 93% spin polarization of the TI surface states from the measured spin signal. In the inverse effect, we inject spin polarized electrons from the Fe into the Bi_2_Te_3_ surface state system, and electrically measure the charge accumulation in the direction orthogonal to that of the injected spin orientation. This provides independent confirmation that spin and momentum are indeed locked in the Dirac surface states of TIs, and more importantly demonstrates versatile electrical access to the TI surface state spin system with important implications for the development of TI-based spintronic devices.

## Results and Discussion

### MBE growth and characterization of Bi_2_Te_3_

**Figure 1:**
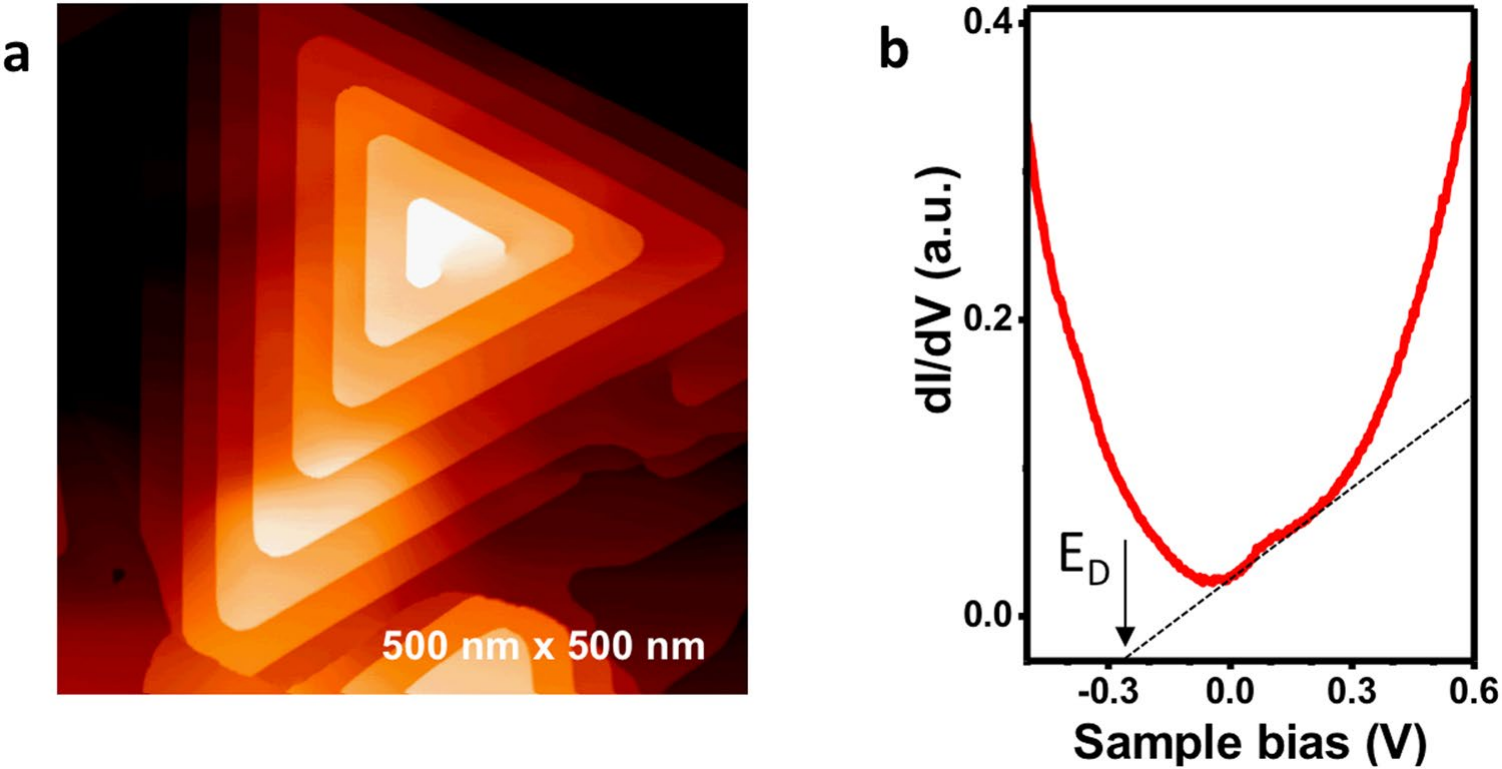
Scanning tunneling microscopy imaging and spectroscopy of MBE grown Bi_2_Te_3_ surface. (**a**) STM image of MBE grown Bi_2_Te_3_ surface taken after growth showing characteristic triangular islands and spiral growth mode. (**b**) Tunneling spectra taken post growth at liquid nitrogen temperature, and extrapolation showing position of the Dirac point (E_D_) to be about 280 meV below the Fermi level (E_F_).

Bi_2_Te_3_ thin films (~15 nm thick) are grown by molecular beam epitaxy (MBE) at 275–325 °C. Epitaxial graphene/SiC(0001) are used as substrates, and Bi and Te fluxes are supplied by Knudsen cells held at 460 and 250 °C, respectively (detailed growth conditions are described in Methods). To facilitate *in situ* scanning tunneling microscopy/spectroscopy (STM/STS) characterization of the sample surface, growth morphology, and electronic structures, a conductive substrate (nitrogen doped *n*-type 6H-SiC (0.1 Ω-cm)) is used. Figure 1a illustrates the layer-by-layer spiral growth characteristics of van der Waals epitaxy due to anisotropic strong in-plane covalent bonding and weak out-of-plane van der Waals bonding^26^. The STS spectra shown in Fig. 1b is taken *in situ* post growth at 77 K, and exhibits a characteristic V-shape with a minimum near 0 V (~−20 meV). Since for Bi_2_Te_3_ the Dirac point is buried within the valence band, this minimal conduction cannot be attributed to the Dirac point. A linear extrapolation method^27^ is used to determine the Dirac point to be ~280 meV below the Fermi level, indicating *n*-type conductivity with the Fermi level well above the conduction band minimum and an estimated carrier concentration of ~9 × 10^12^/cm^2^.

Immediately after the sample is removed from ultrahigh vacuum (UHV), boron nitride (BN) grown by chemical vapor deposition on copper (Graphene Supermarket) was transferred onto the Bi_2_Te_3_ surface using standard polymer (PMMA) based techniques. Boron nitride, a monolayer material with a wide bandgap, has been shown to be a viable tunnel barrier^28^, and can protect the surface from oxidation, and prevent potential interaction between a metal contact and the TI. A 2 nm Al_2_O_3_ tunnel barrier was then grown in a separate MBE system, where an Al deposition + oxidation process was employed (see Methods). The sample was then transferred to an interconnected metals MBE chamber under UHV conditions, where polycrystalline Fe of ~20 nm was deposited from a Knudsen cell at room temperature. We have utilized a similar combination of oxide + monolayer material tunnel barrier, *i.e*., MgO/graphene, for the electrical detection of current-generated spin in Bi_2_Se_3_^10^.

### Device fabrication and measurement geometry

**Figure 2:**
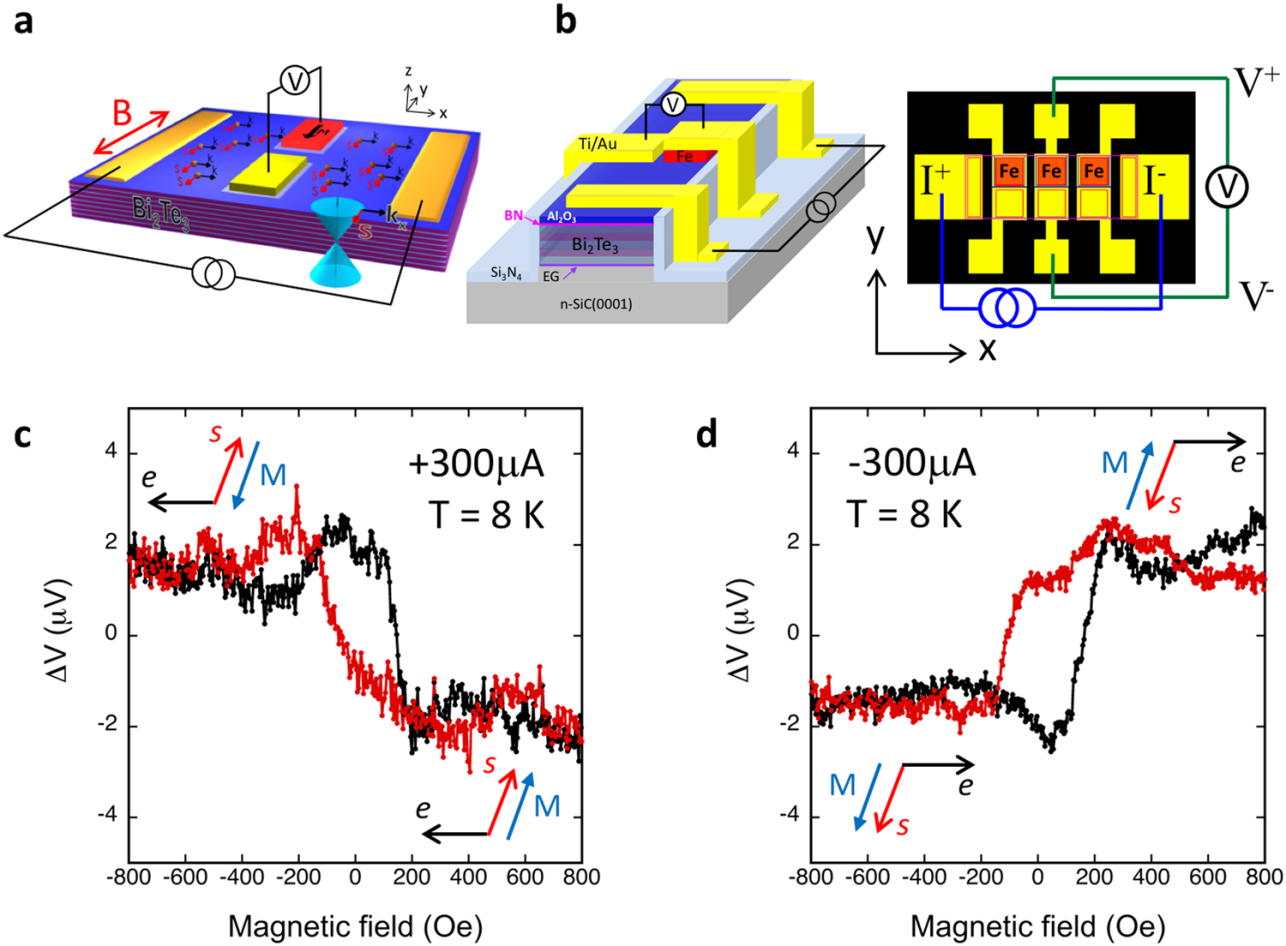
Experimental concept and TI spin polarization detected as a voltage on the Fe/Al_2_O_3_/BN contacts. (**a**) Concept drawing of the transport experiment. (**b**) Top view of contact layout, the top rows (red) of collinear detector contacts is ferromagnetic (Fe), and the bottom row (yellow) is non-magnetic reference contacts (Au/Ti). The ferromagnetic and non-magnetic reference contacts are 80 × 80 μm^2^ with a distance of 45 μm from edge to edge. The gap between the magnetic and corresponding non-magnetic reference contact is 15 μm. (**c**) The ferromagnetic detector contact voltage measured as a function of the applied in-plane magnetic field at 8 K, for bias currents of +300 μA and (**d**) −300 μA.

The samples were then processed, using conventional photolithography techniques, into device structures shown in Fig. 2a,b for transport measurements. A typical device consist of two current leads (Ti/Au) on opposing ends of the Bi_2_Te_3_ mesa, between which a number of ferromagnetic detector (Fe, red) and corresponding non-magnetic reference contact (Ti/Au, yellow) pairs are positioned. When an unpolarized bias current is applied between the two current leads, a spontaneous spin polarization is induced throughout the channel in the Bi_2_Te_3_ surface states, as a result of spin-momentum locking. The projection of this spin onto the magnetization of the ferromagnetic detector contact manifest as a voltage, and is measured with a high-impedance voltmeter (>1 Giga-ohm). Note that the detector contact has no current flowing through it. The magnetization of the detector contact can be switched by an applied in-plane magnetic field, such that it is either parallel or antiparallel to the current generated spins in the TI surface states, hence changing the detector voltage magnitude. Here the positive magnetic field is defined as along the +*y* direction, and positive current as holes flowing in the +*x* direction from left to right.

### Charge-to-spin conversion due to spin-momentum locking

To confirm that the TI samples have the surface states that exhibit spin-momentum locking properties, and that the signals we observe indeed arise from the intrinsic properties of the Dirac surface states of the TI, we first conduct the charge-to-spin conversion measurement to electrically detect the spin generated by an unpolarized current flowing through the surface states.

Figure 2c shows the magnetic field dependence of the Fe/Al_2_O_3_/BN tunnel contact detector voltage for +300 μA current, which generates a spin along the +y direction as a result of spin-momentum locking. At negative magnetic fields greater than the coercive field of Fe, the detector magnetization ***M*** is antiparallel to the TI spin orientation ***s***, and a high voltage is measured. As the field increases to positive values above the ferromagnet coercive field, the detector magnetization is switched to be parallel to the TI spin orientation, and a low voltage value is measured. As the field sweep is reversed from positive to negative, the magnetization of the detector is changed from parallel to antiparallel to TI spin, and the detector voltage switches back from low to high. The hysteretic behavior of the detector voltage is due to the non-zero value of the Fe detector’s coercive field. As the current is reversed to −300 μA, it produces an induced spin in the −y direction (Fig. 2d), and the magnetic field dependence also switches sign (i.e the hysteresis loop flips about the x-axis). A low voltage is again observed when the magnetization and TI spin orientation are parallel, and high voltage when they are antiparallel.

For highly doped n-type Bi_2_Te_3_, band bending is likely to occur on the surface, resulting in carrier accumulation and a coexistence of trivial Rashba 2DEG states, similar to that found on Bi_2_Se_3_^29,30^. The structural inversion asymmetry along the surface normal lifts spin degeneracy via spin-orbit coupling, leading to a pair of Fermi surfaces that exhibit counter-rotating chiral spin texture that also locks spin to the linear momentum. Hence a current in these ordinary 2DEG states can also generate a spin polarization^19,31^. We have found that the current generated spin polarization is opposite for the TI Dirac states and the trivial Rashba 2DEG, and have developed a model to directly derive the sign of the spin voltage expected for the TI surface states^18^. Application of this analysis indicates that the sign of the spin signal observed in Fig. 2c,d is consistent with spin polarization produced by the TI Dirac states rather than the trivial 2DEG surface states.

The spin polarization of the TI surface states can be deduced based on the model calculation from Hong *et al*.^19^. In this work, they find that a three-terminal potentiometric set-up, similar to our measurement geometry, can be used to probe the TI channel polarization, by measuring the voltage change at the ferromagnetic detector contact upon reversing the magnetization^19^. Using a nonequilibrium Green’s function based model, they also derived an analytical expression for the detector voltage that is applicable to both TI surface states and Rashba 2DEG, and in both ballistic and diffusive limits:

1 $$[{V}(+{M})-{V}(-{M})]={{I}}_{{b}}{{R}}_{{B}}{{P}}_{{FM}}({\bf{p}}{{\bf{.M}}}_{{\bf{u}}})$$

where *I*_*b*_ is the (hole) current flowing along +*x*, *R*_*B*_ is the channel ballistic resistance, and *P*_*FM*_ the ferromagnetic detector spin polarization. Here bold case indicates a vector, where **M**_**u**_ is a unit vector along the direction of the detector magnetization **M**, and **p** is the induced spin polarization (per unit current), from both TI Dirac surface states due to spin-momentum locking and trivial 2DEG due to Rashba spin-orbit coupling^19^.

**Figure 3:**
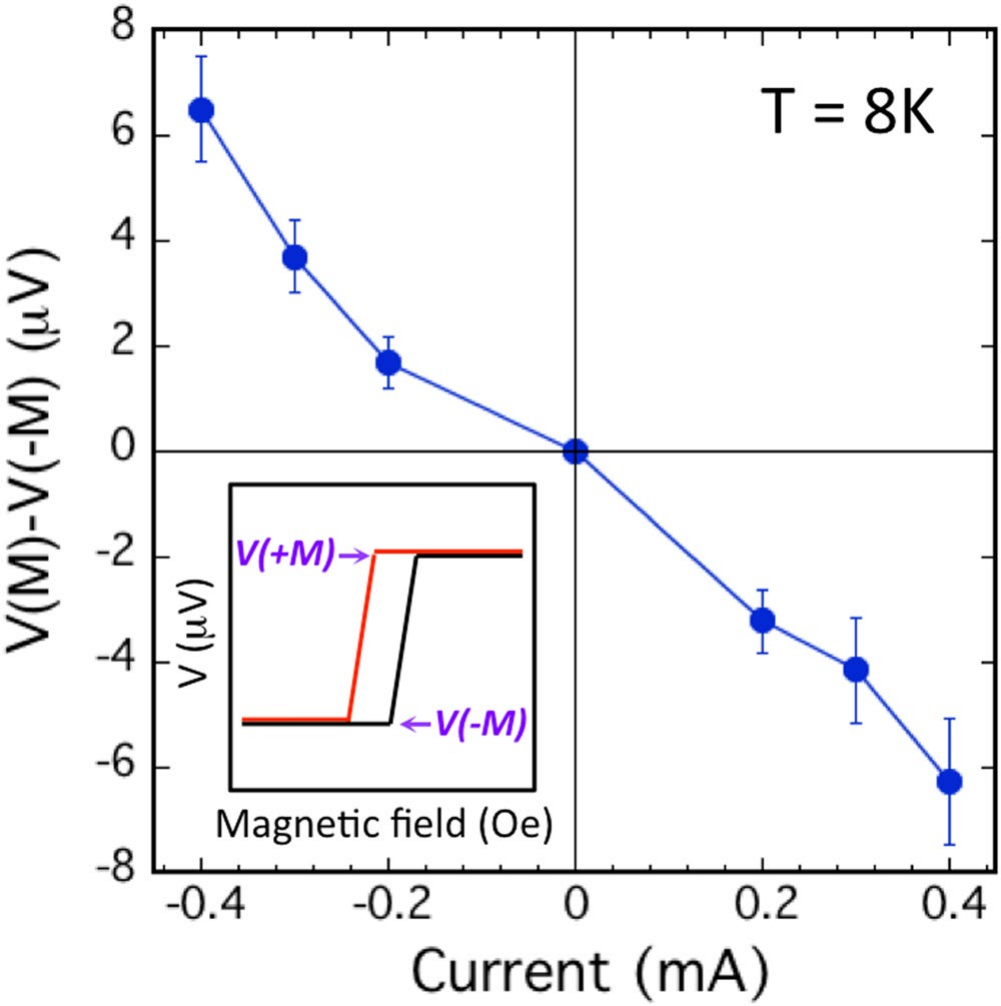
Bias current dependence of the ferromagnetic detector voltage. Amplitude of the ferromagnetic detector voltage hysteresis curve above saturation field of the detector, *ΔV* = *V*(+*M*) − *V*(−*M*), as a function of the bias current at 8 K. The inset illustrates how *ΔV* is determined.

The detector contact spin voltage (*ΔV* = *V*(+*M) − V(−M)*) as a function of the applied current at T = 8 K is shown in Fig. 3, where a nearly linear dependence is observed, consistent with the model calculation discussed above^19^. From the spin signal we measure (e.g., Fig. 3), assuming that the bias current is shunted equally by each quintuple layer of the Bi_2_Te_3_ film^10,17,18^, and the Fe polarization *P*_*FM*_ (Fe) of ~0.4, and *k*_*F*_ of ~0.1Å^−1^^32,33^, we estimate a polarization value **p** of ~−0.93, with a sign that’s indicative of the TI Dirac states^18,19^.

We note that this polarization is significantly higher than the values reported previously for Bi_2_Se_3_^10,18^ and Sb_2_Te_3_^17^ in our studies. We tentatively attribute this to the mechanical transfer of the BN monolayer onto the TI surface immediately after removal from the growth chamber. Such a layer can minimize oxidation and associated degradation of the TI surface, which would otherwise degrade the quality of the tunnel barrier contact and reduce the measured polarization. A more definitive determination of the effect of the additional monolayer BN tunnel barrier on measured polarization would require additional studies where only BN (multilayers) are utilized as the non-hybrid tunnel barrier, which is outside the scope of this study.

### Spin-to-charge conversion due to inverse Edelstein effect

**Figure 4:**
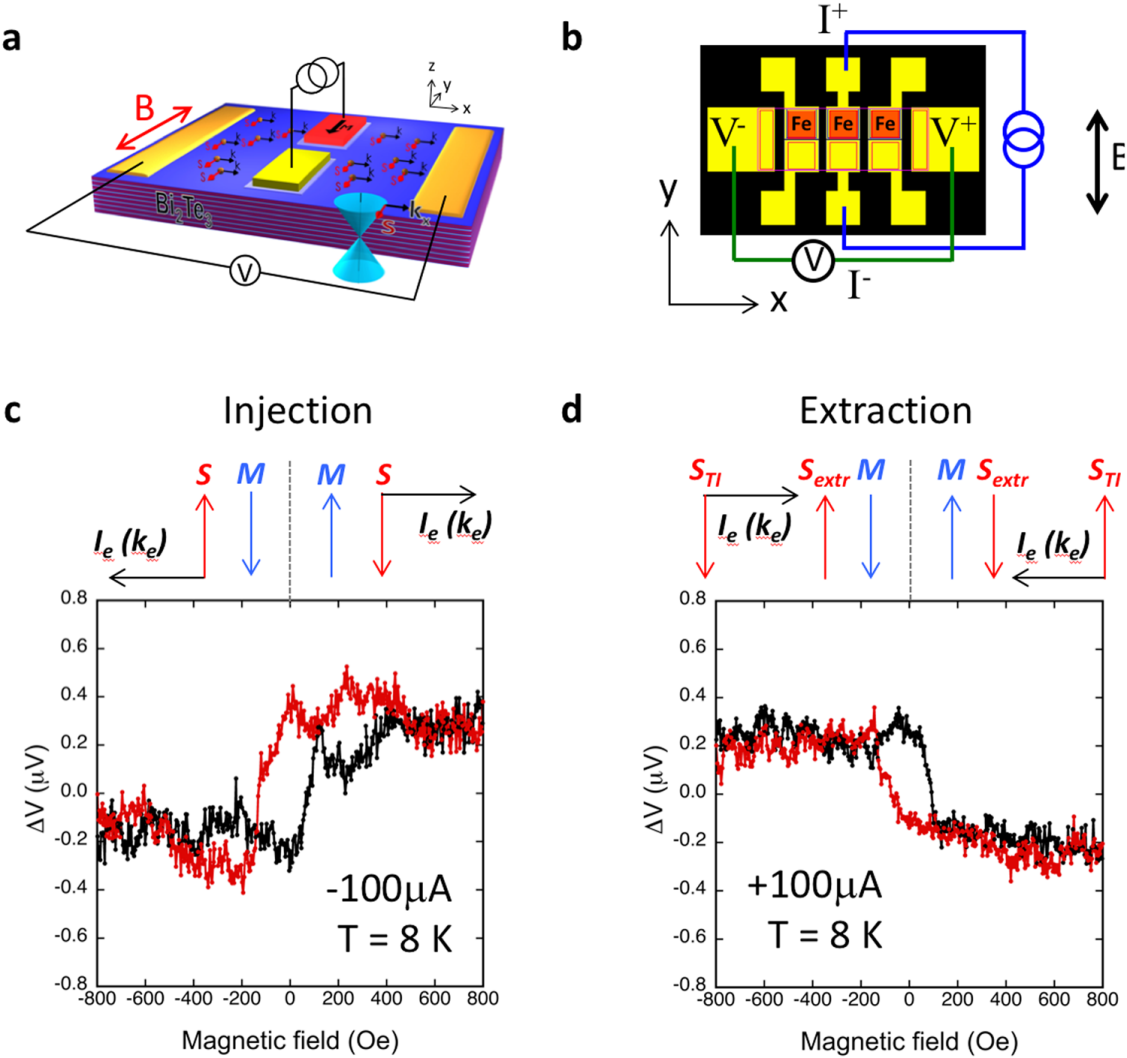
Electrical measurement of spin-to-charge conversion. (**a**) Concept drawing of the spin injection and extraction experiment. (**b**) Contact layout (top view). Voltage measured at adjacent Au/Ti contacts when spin polarized carriers are injected from the ferromagnetic Fe/Al_2_O_3_/BN contact into the TI surface states (**c**), and extracted from the TI surface states into the ferromagnetic contact (**d**) at 8 K.

Next we demonstrate the inverse experiment of spin-to-charge conversion, where a spin polarized electron is injected into the TI surface states. Because of spin-momentum locking, these injected spins should have an associated distinct momentum, and therefore result in a charge accumulation of a certain sign. As shown in Fig. 4a, the same sets of contacts are used, but the voltage and current terminals are switched compared to that of Fig. 2a,b. Specifically, the Fe tunnel contacts are now used as a source of spin-polarized carriers to inject spins into the surface states of the TI, and the Ti/Au contacts on opposing ends of the TI mesa are used as voltage terminals to detect the charge accumulation in the transverse direction.

The magnetization of the ferromagnetic contact, and hence the spin orientation to be injected into the TI, is re-oriented by an applied in-plane magnetic field. For example, with an applied magnetic field in the +y direction (above the saturation field of Fe), (see Fig. 4c arrow diagram above the plot), the magnetization of the Fe contact is also aligned along the +y direction. Since the Fe magnetic moment is opposite to the orientation of its majority spin^34^, the majority spin orientation in the Fe is along −y. When this spin is injected into the TI surface states (Fig. 4c), it should produce an electron momentum in the +x direction due to spin momentum locking. This generates a negative charge accumulation at the +x end of the voltage terminal (labeled V^+^), where a higher voltage is measured compared to that at the V^−^ terminal.

Switching the magnetic field to the −y direction above saturation, and aligning the Fe magnetic moment to −y, enables the injection of spin oriented in the +y direction into the TI surface states. These spins are associated with an electron momentum in the −x direction again due to spin-momentum locking, resulting in a charge accumulation in the −x direction at the V^−^ terminal, and hence a lower voltage should be measured at the V+ terminal.

The plot in Fig. 4c shows the dependence of the voltages measured at the V+ terminal on applied magnetic field. A higher voltage is indeed seen at the +M fields when spins along the −y direction are injected into the TI surface states, and a lower voltage is measured at −M fields when spins along the +y direction are injected. These results clearly demonstrate the spin-to-charge conversion that results from injecting spin polarized electrons into the TI surface states.

Charge accumulation can also be generated via this process by spin extraction, in which one reverses the bias so that spin-polarized electrons flow from the TI surface states into the ferromagnetic metal (Fig. 4d). Qualitatively, one expects the hysteretic loop measured in Fig. 4c to be inverted about the x-axis. As show in the arrow diagram in Fig. 4d (above the plot), when the Fe magnetization direction is oriented in the +y direction by the applied in-plane magnetic field, a majority spin direction along −y is generated in the Fe. Due to the preferential transmission of spins that are also aligned along −y from the TI into the Fe contact, an accumulation of spins along the +y direction remains in the TI surface states. These spins would exhibit an electron momentum along the −x direction due to spin-momentum locking, and generates a charge accumulation in the −x direction at the V− terminal, and hence a lower voltage should be detected at the V+ terminal.

**Figure 5:**
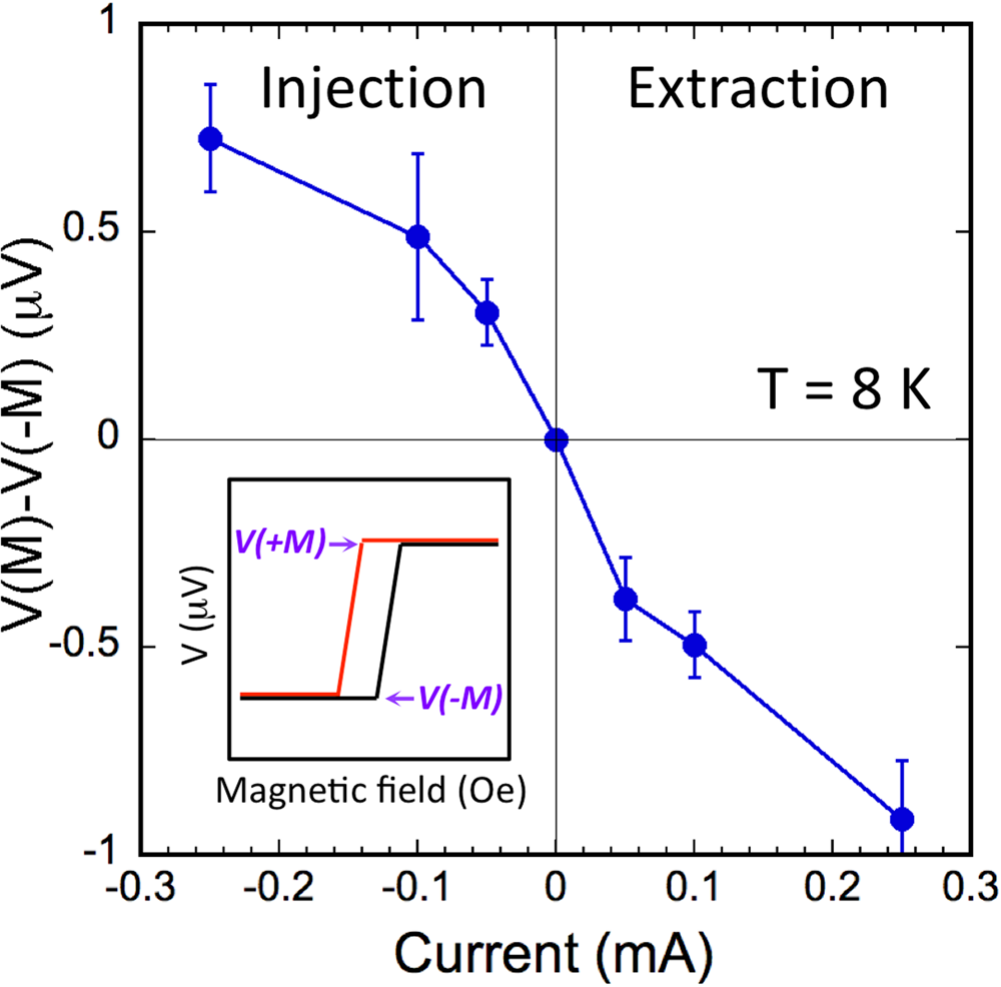
Bias current dependence of the charge accumulation due to spin injection into the TI surface states. Amplitude of the ferromagnetic detector voltage hysteresis curve above saturation field of the detector, *ΔV* = *V*(+*M*)* − V*(*−M*), as a function of the applied bias current at 8 K. The inset illustrates how *ΔV* is determined.

Conversely, when the magnetic field is switched to the −y direction, the Fe magnetic moment is along −y with a majority spin in the +y direction. Hence +y spins are preferentially extracted from the TI into the Fe, leaving a build up of spins along the −y in the TI, which exhibit an electron momentum, and in turn charge accumulation, in the +x direction. This is indeed observed as shown in Fig. 4d for the voltage response as a function of field for +100 μA, further confirming the spin-to-charge conversion in the surface states of the TI. The charge accumulation detected at the V^+^ terminal (*ΔV* = *V*(+*M)* − *V(−M)*) as a function of the injection and extraction current at T = 8 K is shown in Fig. 5, where a monotonic increase in the charge accumulation as a function of the injection and extraction current is observed, as expected.

## Discussion

The common origin for charge-to-spin and spin-to-charge conversion is spin-momentum locking. The fact that spin is locked at right angles to momentum dictates that, given a momentum, i.e., electron motion or charge current, a spontaneous spin polarization should arise. Conversely, given an equilibrium spin population, as achieved by either spin injection from (or spin extraction into) a ferromagnetic source, there must be an electron motion or charge accumulation. The observation of both of these effects as demonstrated here is further evidence that spin and momentum are indeed locked for the surface states of topological insulators, and attest to the robustness of this spin system.

This study is also the first to utilize monolayer BN as part of the Al_2_O_3_/BN hybrid tunnel barrier, which may play a role in the much higher spin polarization observed for the TI surface states than our previous studies on Bi_2_Se_3_^10,18^ and Sb_2_Te_3_^17^. In one of these studies we employed graphene as part of a MgO/graphene hybrid tunnel barrier^10^ where we did not observe such high polarization. Two factors may be at play. First, unlike graphene that’s very conductive in-plane, BN is insulating, and hence can alleviate any current shutting through the monolayer. Second, the BN here was transferred onto the Bi_2_Te_3_ immediate upon removal from the MBE growth camber, whereas the graphene was transferred weeks later onto the TI (Bi_2_Se_3_) for the samples in ref.^10^. This may have more effectively minimized oxidation and associated degradation of the TI surface, which would otherwise degrade the quality of the tunnel barrier contact and reduce the measured polarization. Further work is underway to determine the specific role of the BN tunnel barrier by systematically varying the thickness of the BN, the elapsed time between removal of the sample from vacuum and *ex situ* BN “capping”, and selective post-growth exposure of the TI surface before capping.

In summary, we have demonstrated both charge-to-spin and spin-to-charge conversion in the same Bi_2_Te_3_ based device using a magnetic tunnel barrier contact for electrical spin injection and detection. With an Fe/Al_2_O_3_/BN hybrid tunnel contact, we electrically detect the spin accumulation generated by an unpolarized bias current flowing through the TI surface states. We detect the inverse spin-to-charge conversion process by injecting spins into the Bi_2_Te_3_ surface state system, and electrically measuring a charge accumulation in the orthogonal direction. Analysis of the sign of both the spin and charge accumulation signals indicate that they are consistent with that expected from the TI Dirac surface states. In addition, we find that the use of an h-BN layer on the TI surface provides a significantly higher bias-current induced spin polarization than obtained previously. These results demonstrate the robustness and versatility of electrical access to the TI surface state spin system, and offer additional avenues for spin manipulation, an important step towards the utilization of TIs in next generation spintronic devices.

## Methods

Bi_2_Te_3_ thin films were epitaxially grown on epitaxial graphene/SiC(0001) substrates in an integrated two molecular beam epitaxy (MBE)/low temperature scanning tunneling microscope system with a base pressure of ~1 × 10^−10^ Torr. Epitaxial graphene/SiC(0001) substrates were prepared by first etching as-received N-doped 6H-SiC(0001) substrates in a separate system in H_2_/Ar at an atmospheric pressure at ~1500 °C to remove polishing damages. The SiC substrates were subsequently loaded into the MBE chamber and annealed in UHV, first in Si flux of ~0.1 ML/min at 950 °C to generate a surface with a (3 × 3) reconstruction, and then without Si flux at 1000–1300 °C where epitaxial graphene is grown on the SiC substrate. This technique has been routinely employed in our previous studies to growth epitaxial graphene on SiC^26^. Bi_2_Te_3_ thin films were then grown on this substrate in the same MBE chamber at 275–325 °C, with Bi and Te fluxes provided by individual Knudsen cells held at temperatures 460 and 250 °C, respectively. The samples were transferred into the interconnected STM chamber where the surface morphology was characterized and optimal layer-by-layer spiral growth was verified. Tunneling spectroscopy was also carried out to determine the Dirac point relative to the Fermi level and surface doping, as discussed in Fig. 1(b) in the main text.

A chemical vapor deposited boron nitride (BN) layer is transferred onto the Bi_2_Te_3_ surface immediately upon removal from vacuum, using standard PMMA based transfer techniques. Fe/Al_2_O_3_ tunnel contacts were then grown in a separate MBE system following our established recipe. First, a 0.7 nm layer of polycrystalline Al was deposited at room temperature by MBE, the Al was then oxidized in 200 Torr of oxygen gas in the presence of UV light for 20 minutes in the connected load-lock chamber. These steps were then repeated one more time to reach 2 nm thickness of the Al_2_O_3_. The sample was then transferred to an interconnected metals MBE chamber under UHV conditions, and 20 nm polycrystalline Fe was deposited from a Knudsen cell at room temperature.

The Bi_2_Te_3_ thin film samples were then processed into the device structures shown in Fig. 2a,b using standard photolithography techniques for transport measurements. Fe contacts ranging from 20 × 20 to 80 × 80 μm^2^ were defined by chemical etching methods, with separations between adjacent contacts varying between 45 and 200 μm. The Bi_2_Te_3_ mesas were patterned using ion milling. Ti/Au (10/100 nm) contacts for bias current leads and non-magnetic references were deposited by electron beam evaporation followed by lift off. The Fe contacts were also capped with Ti/Au (10/100 nm). All Ti/Au bond pads are isolated from the SiC substrate by 100 nm Si_3_N_4_.

Transport measurements were carried out in closed cycle cryostat systems with a temperature range of 4–300 K, which are also equipped with an electromagnets that can supply magnetic fields up to ±5000 Oe. In the charge-to-spin measurements, the Ti/Au contacts on opposing ends of the Bi_2_Te_3_ mesa were used to apply unpolarized bias currents, and the ferromagnetic detector contact voltages were measured as a function of the applied in-plane magnetic field perpendicular to the direction of the bias current in the TI, while in the spin-to-charge measurements, the inner ferromagnetic contacts were used to inject (and extract) spins from the TI, and the outer Ti/Au contacts used to detect the charge accumulation.

### Data availability

The datasets generated during and/or analyzed during the current study are available from the corresponding author on reasonable request.

## References

[1] Moore JE (2010). The birth of topological insulators. Nature.

[2] Hasan MZ, Kane CL (2010). Colloquium: Topological insulators. Rev. Mod. Phys..

[3] Fu L, Kane CL, Mele EJ (2007). Topological insulators in three dimensions. Phys. Rev. Lett..

[4] Pesin D, MacDonald AH (2012). Spintronics and pseudospintronics in graphene and topological insulators. Nature Materials.

[5] Kong D, Cui Y (2011). Opportunities in chemistry and materials science for topological insulators and their nanostructures. Nature Chemistry.

[6] Hsieh D (2008). A topological Dirac insulator in a quantum spin Hall phase. Nature.

[7] Zhang H (2009). Topological insulators in Bi_2_Se_3_, Bi_2_Te_3_ and Sb_2_Te_3_ with a single Dirac cone on the surface. Nat. Phys..

[8] Hsieh D (2009). A tunable topological insulator in the spin helical Dirac transport regime. Nature.

[9] McIver JW, Hsieh D, Steinberg H, Jarillo-Herrero P, Gedik N (2012). Nature Nanotechnol..

[10] Li CH (2014). Electrical detection of charge-current-induced spin polarization due to spin-momentum locking in Bi_2_Se_3_. Nature Nanotech..

[11] Tang J (2014). Electrical Detection of Spin-Polarized Surface States Conduction in (Bi_0.53_Sb_0.47_)_2_Te_3_ Topological Insulator. Nano Lett..

[12] Ando Y (2014). Electrical Detection of the Spin Polarization Due to Charge Flow in the Surface State of the Topological Insulator Bi_1.5_Sb_0.5_Te_1.7_Se_1.3_. Nano Lett..

[13] Liu L (2015). Spin-polarized tunneling study of spin-momentum locking in topological insulators. Phys. Rev. B.

[14] Tian J, Miotkowski I, Hong S, Chen YP (2015). Electrical injection and detection of spin-polarized currents in topological insulator Bi_2_Te_2_Se. Sci. Rep..

[15] Lee JS, Richardella A, Hickey DR, Mkhoyan KA, Samarth N (2015). Mapping the chemical potential dependence of current-induced spin polarization in a topological insulator. Phys. Rev. B.

[16] Dankert A, Geurs J, Kamalakar MV, Dash SP (2015). Room Temperature Electrical Detection of Spin Polarized Currents in Topological Insulators. Nano Lett..

[17] Li CH, van ‘t Erve OMJ, Li YY, Li L, Jonker BT (2016). Electrical Detection of the Helical Spin Texture in a p-type Topological Insulator Sb_2_Te_3_. Sci. Rep..

[18] Li CH, van ‘t Erve OMJ, Rajput S, Li L, Jonker BT (2016). Direct comparison of current-induced spin polarization in topological insulator Bi_2_Se_3_ and InAs Rashba states. Nat. Commun..

[19] Hong S, Diep V, Datta S, Chen YP (2012). Modeling potentiometric measurements in topological insulators including parallel channels. Phys. Rev. B..

[20] Edelstein VM (1990). Spin polarization of conduction electrons induced by electric current in two-dimensional asymmetric electron systems. Solid State Commun..

[21] Shiomi Y (2014). Spin-electricity conversion induced by spin injection into topological insulators. Phys. Rev. Lett..

[22] Kondou K (2016). Fermi-level-dependent charge-to-spin current conversion by Dirac surface states of topological insulators. Nat. Phys..

[23] Wang H (2016). Surface-State-Dominated Spin-Charge Current Conversion in Topological-Insulator–Ferromagnetic-Insulator Heterostructures. Phys. Rev. Lett..

[24] Rojas-Sánchez J-C (2016). Spin to charge conversion at room temperature by spin pumping into a new type of topological insulator: α-Sn films. Phys. Rev. Lett..

[25] Song Q (2016). Spin injection and inverse Edelstein effect in the surface states of topological Kondo insulator SmB6. Nat. Commun..

[26] Liu Y, Weinert M, Li L (2012). Spiral growth without dislocations: molecular beam epitaxy of the topological insulator Bi_2_Se_3_ on epitaxial graphene/SiC(0001). Phys. Rev. Lett..

[27] Zhu X-G (2016). Electronic structures of topological insulator Bi_2_Te_3_ surfaces with non-conventional terminations. New J. Phys..

[28] Britnell L (2012). Electron tunneling through ultrathin boron nitride crystalline barriers. Nano Lett..

[29] Bahramy MS (2012). Emergent quantum confinement at topological insulator surfaces. Nat. Commun..

[30] King PDC (2011). Large Tunable Rashba Spin Splitting of a Two-Dimensional Electron Gas in Bi_2_Se_3_. Phys. Rev. Lett..

[31] Bychkov YA, Rashba EI (1984). Properties of a 2D electron gas with lifted spectral degeneracy. JEPT Lett..

[32] Wang G (2011). Topological insulator thin films of Bi_2_Te_3_ with controlled electronic structure. Adv. Mat..

[33] Li YY (2010). Intrinsic topological insulator Bi_2_Te_3_ thin films on Si and their thickness limit. Adv. Mat..

[34] Jonker BT, Hanbicki AT, Pierce DT, Stiles MD (2004). Spin nomenclature for semiconductors and magnetic metals. J. Magn. Magn. Mater..

